# A Miniaturized and Highly Stable Frequency-Selective Rasorber Incorporating an Embedded Transmission Window

**DOI:** 10.3390/mi15080980

**Published:** 2024-07-30

**Authors:** Yi Li, Yuxi Zhong, Minrui Wang, Keqing Chen, Peng Ren, Zheng Xiang

**Affiliations:** The State Key Laboratory of Integrated Services Networks, Xidian University, Xi’an 710071, China; 15536903769@163.com (Y.Z.); mrwang614@163.com (M.W.); 23011210627@stu.xidian.edu.cn (K.C.); pren@xidian.edu.cn (P.R.); zhx@mail.xidian.edu.cn (Z.X.)

**Keywords:** frequency-selective rasorber (FSR), embedded transmission window, miniaturized structure, broadband absorption, high-stability characteristics

## Abstract

In this article, a miniaturized and highly stable frequency-selective rasorber (FSR) incorporating an embedded transmission window is designed. This FSR consists of a lossy layer loaded with resistors, an air layer, and a bandpass layer. The lossy layer is provided with a rectangular, square ring structure loaded with four 180 Ω resistors and four quadrilateral metal plates. The four metal plates are connected to the four corners of the inner ring around the square ring and are radially distributed along the diagonal. The bandpass layer is a square metal patch that a cross-ring slot structure is loaded inside of, and the cross points lie in the direction along the diagonal of the unit. The inner boundary of the cross-ring is composed of two mutually perpendicular and long rectangular elements. This FSR shows an embedded transmission window from 3.63 GHz to 3.80 GHz and has a transmission rate of 93% at 3.72 GHz. Moreover, both sides of the transmission band, namely, 1.86–3.35 GHz and 3.99–8.28 GHz, have an absorption rate of more than 80% and bilateral relative bandwidth of more than 50%. In addition, this structure exhibits excellent miniaturization performance, polarization insensitivity, and angular stability. Finally, a prototype of the designed FSR is processed and measured. The measured results are basically consistent with the simulation results.

## 1. Introduction

The rapid advancement of radar-detection technology and the growing complexity of the electromagnetic environment have disrupted traditional combat systems, posing threats to our battlefield positions and high-value targets. This has resulted in increasingly pressing demands for enhanced anti-detection and stealth capabilities, as well as for comprehensive strategies for communication systems. A frequency-selective surface (FSS) is capable of transmitting or reflecting incident electromagnetic waves in space; hence, it is termed a spatial filter [1,2,3]. With its excellent filtering characteristics, it is widely used in various fields, such as communication, optics, and stealth technology [4,5,6,7,8]. In the early days of single-base-station military detection, an FSS reduced the radar cross-section by reflecting electromagnetic waves in other directions to achieve stealth. However, traditional microwave absorbing materials, when used to achieve full-band stealth, can compromise the antenna’s radiation performance. Against this backdrop, the frequency-selective rasorber (FSR) was introduced as an enhanced structure built upon the traditional FSS technology, incorporating lumped components, active components, metamaterials, etc. [9,10,11,12,13]. The FSR is designed to address the detection challenges posed by multistatic radars in a multidomain battlefield environment by absorbing and dissipating electromagnetic wave energy within the antenna’s nonoperating frequency bands rather than reflecting it outward. This approach minimizes the antenna’s radar cross-section (RCS) in all directions outside its passband, enhancing its stealth capabilities. The primary objective of surface invisibility is to reduce or eliminate the reflection of electromagnetic waves from an object, achieving a stealth effect, whereas the purpose of electromagnetic energy absorption capability is to convert the incident electromagnetic wave energy into other forms of energy [14,15].

Currently, some international researchers have conducted profound studies on FSRs. For example, Ref. [16] studied and discussed the design method of an FSR in detail. The electromagnetic characteristics of the new FSR radome are high-frequency wave absorption and low-frequency wave transmission. Ref. [17] designed an FSR based on windmill-shape coupling line arrays, which have bilateral, narrow absorbing bands on the passband edges. In the same year, the authors of [18] published an FSR structure that demonstrated the fact that the absorption zone and the transmission zone are both bilateral. The frequency range of the absorption zone is 2.1–4.2 GHz and 5.5–12.3 GHz, and the minimum insertion loss is less than 1 dB in the two transmission bands based on an absorption rate of more than 90% in the case of vertical incidence. Moreover, Ref. [19] proposed a dual-polarized frequency-selective rasorber (FSR) with a wide high-transmission passband and two absorption bands, from which the 1 dB transmission window can be obtained from 8.12 to 11 GHz with a 30.4% fractional bandwidth, and the bandwidth of reflection below −10 dB is up to 111%, from 4 to 14 GHz. Ref. [20] designed a dual-polarization FSR with one transmission band and two absorbing bands, for which the center frequency of the passband is 5.74 GHz, with an insertion loss of 0.25 dB. The lower and higher absorption bandwidths with an absorption coefficient of higher than 80% range from 1.92 to 3.73 GHz and from 7.41 to 9.34 GHz, respectively; the reflection band with a reflection coefficient of less than −10 dB ranges from 1.96 to 9.32 GHz. In 2019, a miniaturized high-selectivity FSR with subwavelength resonance and an interdigital resonator was investigated, with a fractional bandwidth for −10 dB reflection of about 94.5%, and the −3 dB transmission relative bandwidth was 15.7% [21]. In 2021, Ref. [22] proposed an FSR with an anisotropic transmission band. When the incident electromagnetic wave is TE-polarized, the transmission band is located at 10.4 GHz, and the absorption rate is greater than 80% in the frequency range of 6.3–9.6 GHz and 11.5–14.1 GHz. Ref. [23] presented a novel FSR with one highly selective passband between two absorption bands, for which the operating bandwidth of the proposed rasorber is 110.6%. Additionally, Ref. [24] showed a dual-polarized rasorber with an inter-absorption transmission band, for which the reflection coefficient below −10 dB is from 2.89 to 8.85 GHz, corresponding to a fractional bandwidth of 101.5%.

While considerable progress has been made in FSR research, the prevalent FSR designs encounter difficulties, such as a low transmission rate and asymmetric absorption bands on either side. This article proposes a miniaturized and highly stable FSR incorporating an embedded transmission window that can achieve a transmission rate of 93% at 3.72 GHz and a suppression reflection rate of less than 20% over a frequency range of 1.86–8.28 GHz. Moreover, on both sides of the transmission band, specifically from 1.86 to 3.35 GHz and 3.99 to 8.28 GHz, the absorption rate exceeds 80%, with bilateral relative bandwidths exceeding 50%. With its excellent filtering characteristics, this FSR can not only be applied in the field of stealth technology to reduce the RCS of targets, thereby enhancing their survivability, but can also be used in the field of electromagnetic compatibility to minimize electromagnetic wave interference between different devices and ensure the normal operation of electronic equipment.

## 2. Analysis and Design of FSR Structure

### 2.1. Theoretical Analysis of FSR

FSR has two main design goals: on the one hand, it aims to reduce the insertion loss of the transmission band; on the other hand, the loss rate of the absorption band should exceed 80%. This article analyzes the achievable electromagnetic performance of the proposed FSR. The FSR needs to not only have bandpass transmission characteristics similar to an FSS but also wave absorption characteristics. Therefore, the design steps of the passband embedded frequency-selective rasorber can be divided into the following three steps: first, design the bandpass frequency-selective surface at the bottom layer to make its passband fall on the required transmission window frequency; second, design the loss surface of the upper layer to achieve the wave absorption effect on the required absorption band, and select the dielectric substrate with an appropriate dielectric constant and thickness. Finally, the quasi-Newton algorithm in HFSS 19.0 software is used to optimize the parameters, such as the thickness of the dielectric substrate, dielectric constant, metal length, the metal width of the absorption layer and the bandpass layer, to achieve the matching of structural surface impedance and free space wave impedance. Ultimately, the target filtering performance metrics, such as absorption rate, absorption bandwidth, transmission bandwidth, and transmission rate, are achieved.

**Figure 1:**
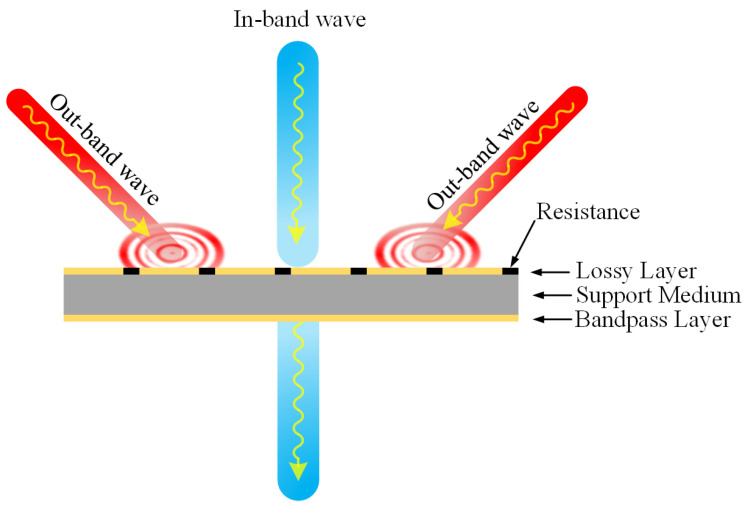
Structure diagram of the FSR.

The FSR is composed of a stacked lossy layer surface, an air layer (in actual production, the air layer is filled with foam material with a dielectric constant close to air), and a bandpass frequency-selective surface arranged from top to bottom. Typically, the transmission line model is used to conduct an equivalent analysis of the overall structure’s filtering characteristics. Figure 1 shows the structure diagram of the FSR structure. It can be seen that the FSR is composed of an upper lossy layer with resistance, a middle support medium, and a lower bandpass layer. It has the ability to transmit a specific in-band wave and absorb the waves of an out-band.

**Figure 2:**
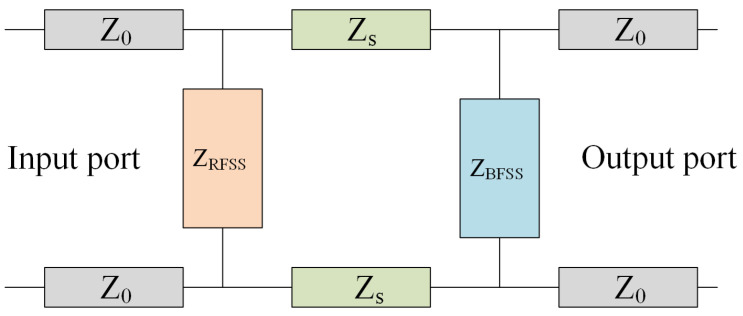
Equivalent circuit of a general FSR.

Figure 2 shows its equivalent transmission line model, where $Z_{0}$ is the characteristic impedance in free space. The main function of the bandpass layer in the FSR is to achieve in-band wave transmission with a characteristic impedance of $Z_{BFSS}$. The lossy layer can absorb out-of-band electromagnetic waves reflected back from the bandpass layer with a characteristic impedance of $Z_{RFSS}$. The supporting medium is mainly used to connect the bandpass layer and the lossy layer, and it plays a role in adjusting the characteristic impedance of the entire FSR structure, with a characteristic impedance of $Z_{s}$ and the relative permittivity of the material $\varepsilon_{r}$.

**Figure 3:**
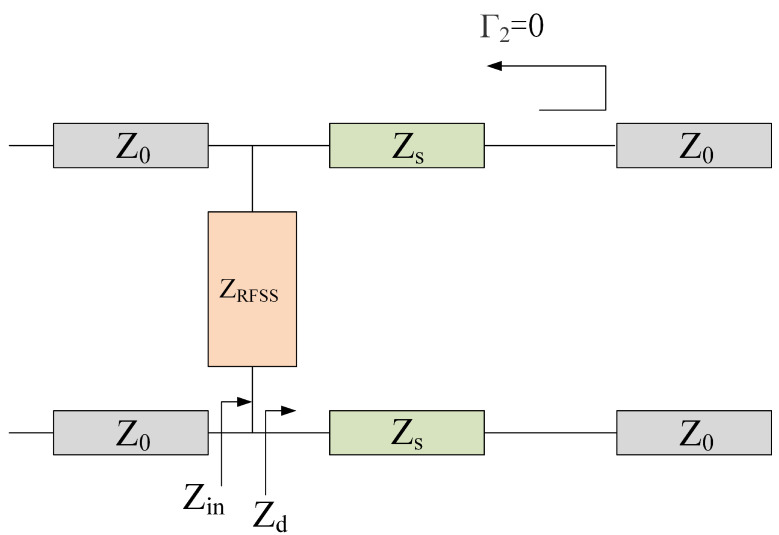
Equivalent circuit under FSR total transmission condition.

**Figure 4:**
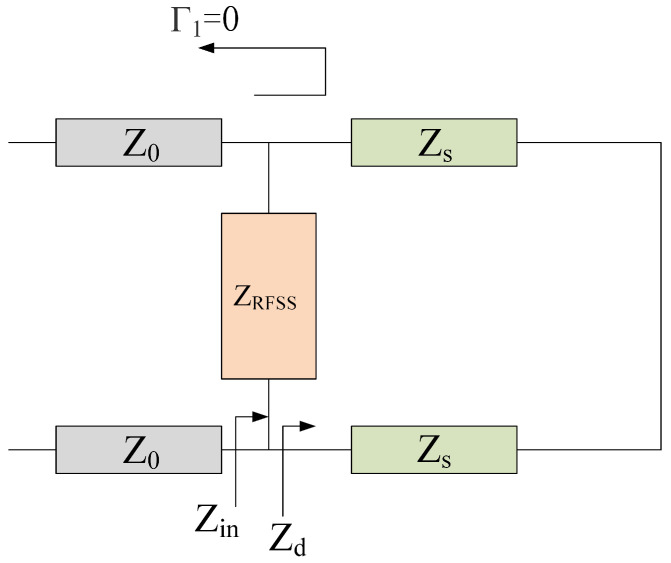
Equivalent circuit under FSR total absorption condition.

In the ideal scenario, when the incident frequency aligns with the transmission band of the FSR, the electromagnetic wave can pass through both the impedance FSS and the bandpass FSS completely; when the incident frequency falls within the absorption band of the FSR, the lower FSS exhibits metal-like total reflection, and the reflected wave is absorbed by the upper impedance frequency-selective surface. It is evident that impedance FSS and bandpass FSS have distinct operational requirements for varying incident frequencies. The equivalent circuit models depicted in Figure 3 and Figure 4 were derived from the discussion of the different filtering performances of the FSR in the absorption and transmission bands.

In an ideal state, if the incident frequency, *f*, is consistent with the transmission frequency, $f_{p}$, of the FSR, the circuit of the bandpass frequency-selective surface at the lower layer is equivalent to an open circuit; therefore, these electromagnetic waves can be completely transmitted. According to the transmission line theory, up to the interface between the impedance frequency-selective surface and the free space of the air layer, the reflection coefficient is

(1) $$\Gamma_{1}=\frac{Z_{\mathrm{in}}-Z_{0}}{Z_{0}+Z_{in}}$$

where the $Z_{in}$ expression is

(2) $$Z_{in}=\frac{Z_{RFSS}Z_{d}}{Z_{d}+Z_{RFSS}}$$

Similarly, when it is in an ideal state, if the incident wave frequency, *f*, is near the absorption frequency, $f_{a}$, of the FRS, the reflection coefficient, $\Gamma_{1}$, is 0 at the interface between the lossy layer FSS and the free space below it, which means that impedance matching and the incident wave is absorbed completely without reflection. Therefore, the characteristic impedance of the lossy layer FSS meets the following formula:

(3) $$Z_{RFSS}=\frac{Z_{0}Z_{d}}{Z_{d}-Z_{0}}$$

In the absorption band, a bandpass FSS is equivalent to a short circuit, so there are

(4) $$Z_{d}=jZ_{s}\tan\left(2\pi df_{a}\sqrt{\varepsilon_{r}}/c\right)$$

(5) $$Z_{RFSS}=\frac{Z_{0}Z_{s}\tan\left(2\pi df_{a}\sqrt{\varepsilon_{r}}/c\right)}{Z_{s}\tan\left(2\pi df_{a}\sqrt{\varepsilon_{r}}/c\right)+jZ_{0}}$$

In conclusion, at the wave transmission frequency, $f_{p}$, the bandpass FSS should allow electromagnetic waves to be transmitted completely; that is, the reflection coefficient, $\Gamma_{2}$, is also 0 at the interface between the bandpass FSS and the support medium/air layer:

(6) $$\Gamma_{2}=\frac{Z_{0}-Z_{s}}{Z_{0}+Z_{s}}=0$$

It is deduced that

(7) $$Z_{s}=Z_{0}$$

That is, the relative permittivity of the support medium is equal to the relative permittivity of the free space, and both need to be consistent. By introducing Formula (7) into Formula (5), the characteristic impedance of the FSS of the lossy layer can be simplified as

(8) $$Z_{RFSS}=\frac{Z_{0}\tan\left(2\pi df_{a}\sqrt{\varepsilon_{r}}/c\right)}{\tan\left(2\pi df_{a}\sqrt{\varepsilon_{r}}/c\right)+j}$$

Formula (8) is the characteristic impedance of the lossy layer FSS near the absorption frequency point, $f_{a}$. If the thickness of the dielectric substrate, *d*, is not equal to one-fourth of the conduction wavelength, then the characteristic impedance will not be a real number; that is, the frequency-selective surface of the lossy layer contains both the real resistance component and the imaginary reactance component. When *d* is exactly equal to one-fourth of the conduction wavelength, $Z_{RFSS}=Z_{0}$, the characteristic impedance of the lossy layer FSS will be a real number, which means pure resistance, and this is numerically equal to the characteristic impedance of free space.

The above analysis shows that the FSR can increase the frequency range of impedance matching by changing the imaginary part of the upper lossy layer FSS characteristic impedance. When compared with the Salisbury absorbing screen, it can achieve a wider absorption band, and the thickness of the supporting dielectric layer is not limited by the operating wavelength.

According to the above basic impedance analysis of the equivalent transmission line model of the FSR, for the incident wave in the absorption and transmission frequency bands, the following conclusions can be drawn about the FSR characteristic impedance: when the incident wave frequency is located at the absorption frequency of the FSR, the lower bandpass FSS is equivalent to a short circuit of total reflection (or metal plate), and the impedance of the lossy layer frequency-selective surface meets Formula (8). If the incident wave frequency is located at the transmission frequency of the FSR, the bandpass frequency-selective surface can be equivalent to an open circuit, and the relative dielectric constant of the middle dielectric layer needs to be consistent with the free space.

### 2.2. Structure Design of the FSR

**Figure 5:**
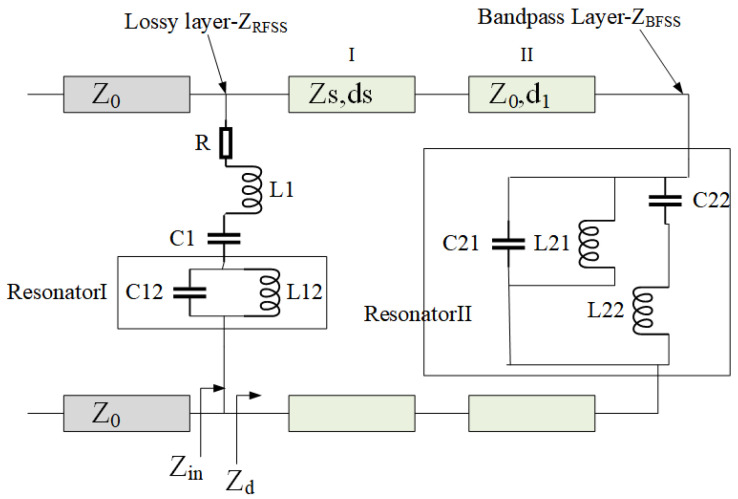
Equivalent circuit model of the proposed FSR in this article.

The ECM of our proposed FSR is shown in Figure 5. Resonator I and resonator II are introduced into the lossy layer and lossless layer, respectively. In this structure, when two resonators resonate at $f_{p1}$, the passband of a single transmission frequency point is realized. According to the transmission line theory, when the incident wave frequency is the wave transmission frequency, the lower lossless layer FSS should facilitate maximum electromagnetic wave transmission; that is, the reflection coefficient is 0 at the interface between the bandpass layer FSS and the support medium or air layer, so the dielectric constant of the dielectric layer above the bandpass layer matches the lower dielectric layer. When the incident wave frequency is in the absorption band ($f_{a1}$ and $f_{a2}$) at both ends of the passband, the impedances of the two resonators are inductive or capacitive. Specifically, $f_{p1}$ is determined by $C_{12}$, $L_{12}$, $C_{21}$, and $L_{21}$, $f_{a1}$ and $f_{a2}$ are determined by *R*, $L_{1}$, $C_{1}$, $C_{22}$, and $L_{22}$. Additionally, $f_{p1}$ can be represented as follows:

(9) $$f_{p1}=\frac{1}{2\pi \sqrt{C_{12}L_{12}}}=\frac{1}{2\pi \sqrt{C_{21}L_{21}}}$$

**Figure 6:**
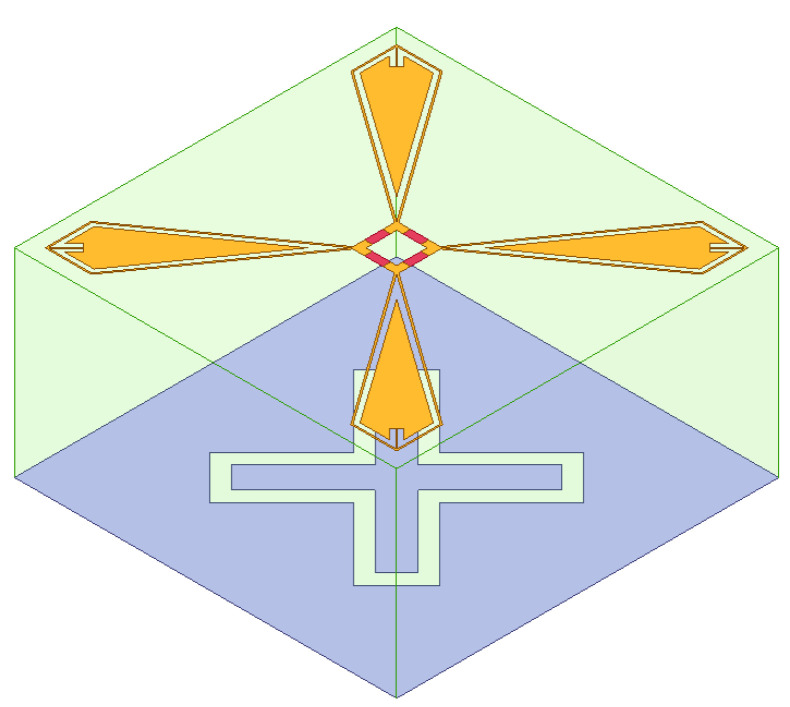
The three-dimensional unit structure diagram of the proposed FSR.

The designed FSR consists of a lossy layer loaded with resistors, an air layer, and a single bandpass FSS as a lossless layer. The three-dimensional structure of its units is shown in Figure 6. The lossy layer is provided with a rectangular, square ring structure loaded with four 180 Ω resistors and four quadrilateral metal plates. The four metal plates are connected with the four corners of the inner ring around the square ring and are radially distributed along the diagonal.

The foundation of the second metal layer is an all-metal plate on which a cross-ring slot is set, and the cross direction lies along the diagonal direction. The center of the rectangular square ring structure, the center of the community composed of four radial metal plates, and the geometric center of the cross-ring slot of the second metal layer coincide with the center of the overall unit, and the overall unit is a centrosymmetric structure. The lossy layer frequency-selective surface and the metal layer are printed on the PCB board as type F4BM-2, and its dielectric constant is 2.65. The thickness of the air layer in the space from the dielectric plate to the metal plate is 12 mm. The distance between the first metal layer and the second metal layer is 13 mm in total. The air layer in the simulation model can be filled with polymethylacrylimide foam called PMI to enhance the mechanical strength of the structure. The dielectric constant of the PMI foam material is 1.07, and the loss tangent is 0.0018.

**Figure 7:**
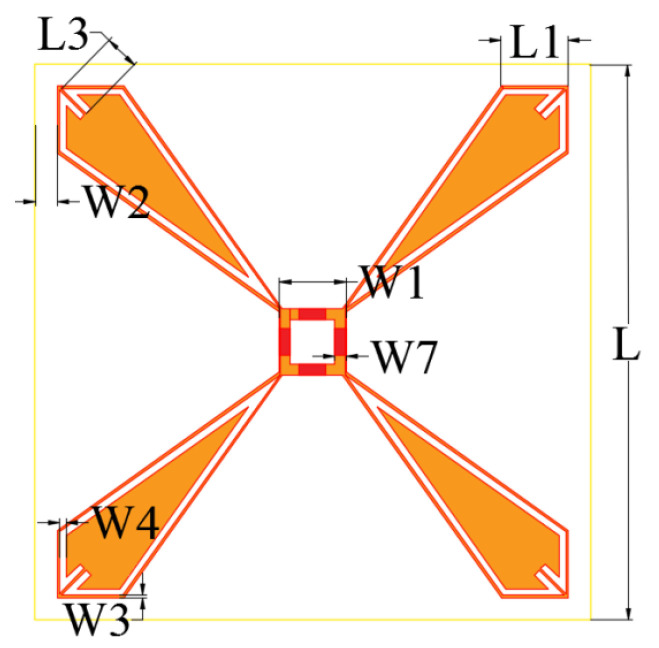
Detailed dimensions of the lossy layer in the FSR unit cells.

**Figure 8:**
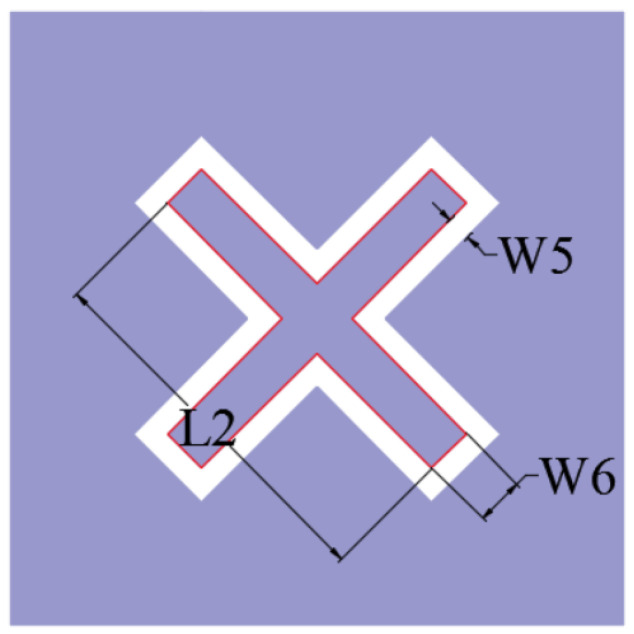
Detailed dimensions of the bandpass layer in the FSR unit cells.

The plane structure of the upper lossy FSS unit is shown in Figure 7. The overall outer boundary of the unit is a square structure with a side length of L=25 mm. A square ring patch is loaded inside, and its center coincides with the overall center of the unit. We load a 0402 chip-fixed resistor at the midpoint of the four sides of the square ring patch, R=180 Ω. A quadrilateral metal patch is, respectively, loaded onto the square ring patch along four diagonal directions. The four quadrilateral metal patches are two long sides with equal length and two short sides with equal length. The short side is parallel to the whole four sides of the unit. The metal patches are radially distributed along the diagonal of the metal layer. The metal plate is loaded with a strip resonant slot aperture structure to achieve the bandpass effect of the lossy layer, which is symmetrically distributed along the diagonal mirror surface of the unit, extending from the position close to the top of the square ring patch in the direction parallel to the long side of the metal sheet; this then turns to the direction parallel to the edge of the structural unit to extend to the top of the metal sheet. The structure of the lower lossless layer FSS unit is shown in Figure 8. The unit is a square metal patch, and a cross-ring slot structure is loaded inside; the cross points to the direction along the diagonal of the unit. The inner boundary of the cross-ring is composed of two mutually perpendicular long rectangular elements. The detailed structural parameters are listed in Table 1. This structure exhibits excellent miniaturization performance; the length and width of a unit are only $0.31\lambda_{T}\times 0.31\lambda_{T}$, where $\lambda_{T}$ represents the wavelength corresponding to the center frequency of the transmission band.

## 3. Analysis of Simulation Results

**Figure 9:**
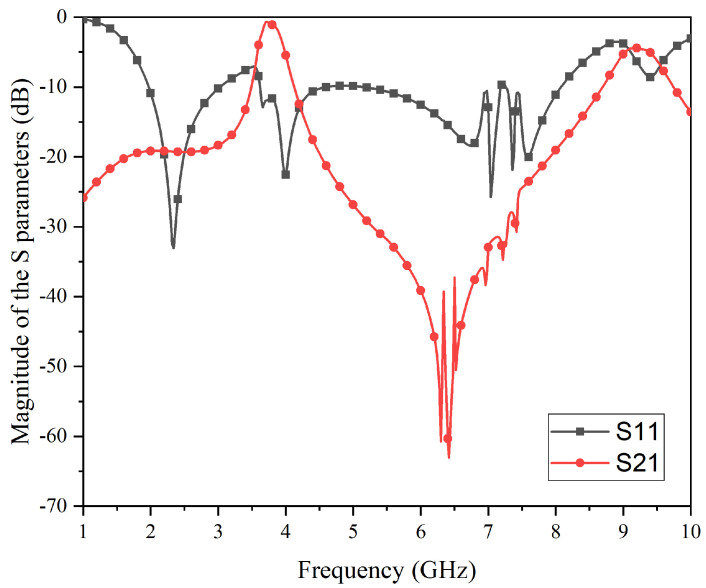
Simulation results for the S-parameters of the proposed FSR.

**Figure 10:**
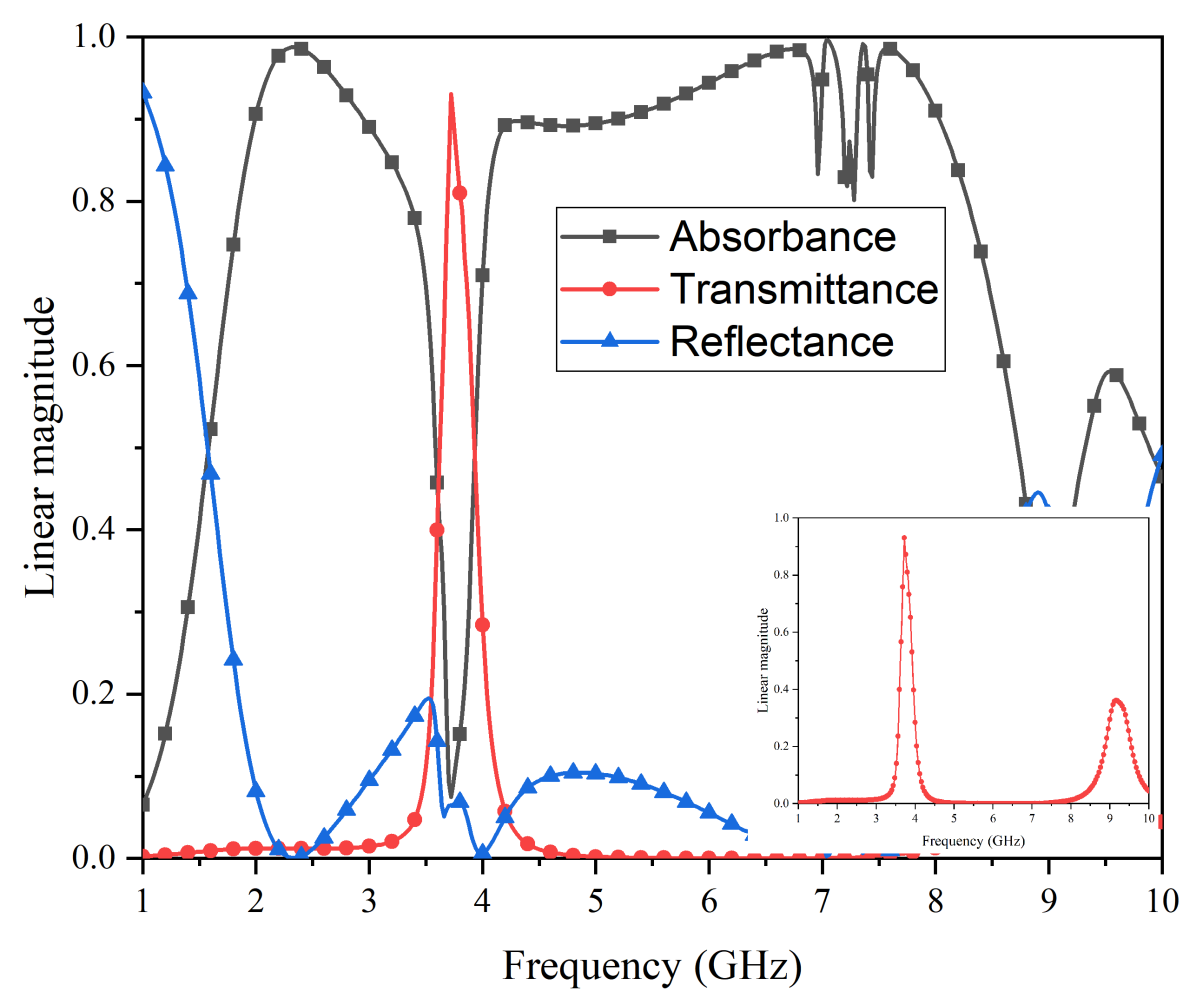
Simulation results for the absorbance, transmittance, and reflectance of the proposed FSR.

This study used commercial simulation software (HFSS 19.0) for the simulation analysis. Figure 9 shows the simulation results of the S-parameters of the FSR under vertical incidence. It can be seen that the $S_{21}$ coefficient near the passband increases to close to 0, while the $S_{21}$ on both sides is suppressed below −20 dB, and the $S_{11}$ is basically suppressed below −10 dB. Figure 10 shows the simulation results of the absorbance (*A*), transmittance (*T*), and reflectance (*R*) for the proposed FSR. The *A*, *T*, and *R* can be obtained as follows: $R={\left|S\left(11\right)\right|}^{2}$; $T={\left|S\left(21\right)\right|}^{2}$; $A=1-{\left|S\left(11\right)\right|}^{2}-{\left|S\left(21\right)\right|}^{2}$. In addition, the method obtained from HFSS is as follows: “Result-Create Modal Solution Data Report-Rectangular Plot-Output Variables”. Then, by inputting the above formulas, the *A*, *T*, and *R* of the curves can be obtained. In addition, the equivalent parameters of the elements and the loss parameters of the materials were all set to the model of the HFSS. Therefore, the EM energy captured by the resistive part of the elements and losses in all materials are all taken into account in the simulations.

According to the requirement of the passband insertion-type FSR, the transmission rate must meet Tra≥80%; the frequency-selective rasorber structure has an excellent transmission rate in the frequency band from 3.63 GHz to 3.80 GHz. The central frequency point of the passband is 3.72 GHz, and the maximum transmissivity is 93%. Based on the reflection rate, Ref≤20%, the reflection rate can be suppressed below 20% in the broadband from 1.86 GHz–8.28 GHz so that it hardly affects the wave absorption/transmission effect. Based on the absorption rate, Abs≥80%, this structure has excellent wave absorption characteristics of more than 80% on both sides of the wave transmission band, namely, the 1.86–3.35 GHz band and the 3.99–8.28 GHz band. The maximum absorption rate reaches 98%, and the central frequencies of the absorption bands are situated at 2.60 GHz and 6.14 GHz, respectively. Furthermore, the relative absorption bandwidths are 57.2% and 69.9%, both surpassing the 50% threshold. Although ripples are observed near 7 GHz, they remain sufficient enough to ensure an adequate relative bandwidth within the absorption band, thereby achieving the anticipated objective of embedding the passband within a broad absorption spectrum. The main reason for the occurrence of ripples is that the upper structure is a centrally connected structure, and the structural coupling characteristics intensify on the resonant path corresponding to 6.75–7.75 GHz.

**Figure 11:**
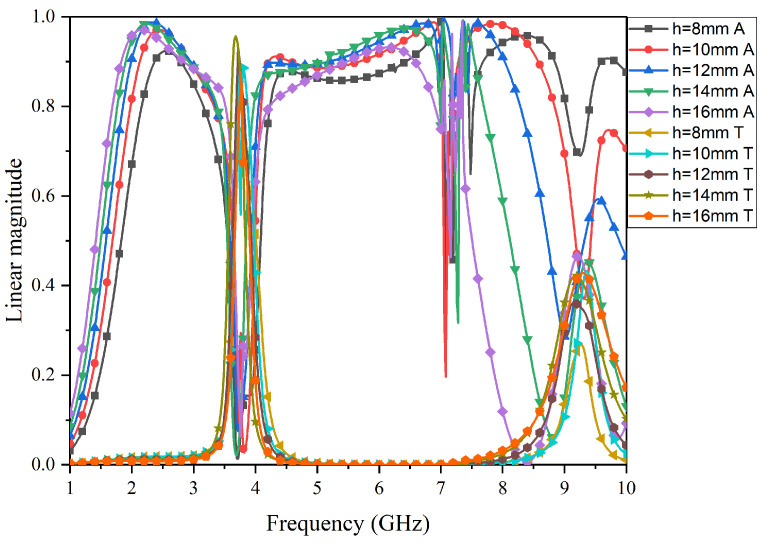
Impact of different air layer thicknesses on FSR filtering performance.

The simulation in Figure 11 shows the impact of the thickness of the air layer on the structural passband and absorption band. As can be seen from the figure, when the thickness is less than 12 mm, the absorption bandwidth decreases, and the passband shifts towards lower frequencies. When the thickness is greater than 12 mm, the absorption bandwidth increases, but the ripples in the high-frequency absorption band become more pronounced. The passband shifts towards higher frequencies, and the transmission rate decreases. Therefore, 12 mm is the optimal dimension for achieving the best absorption and transmission performance of this FSR structure.

**Figure 12:**
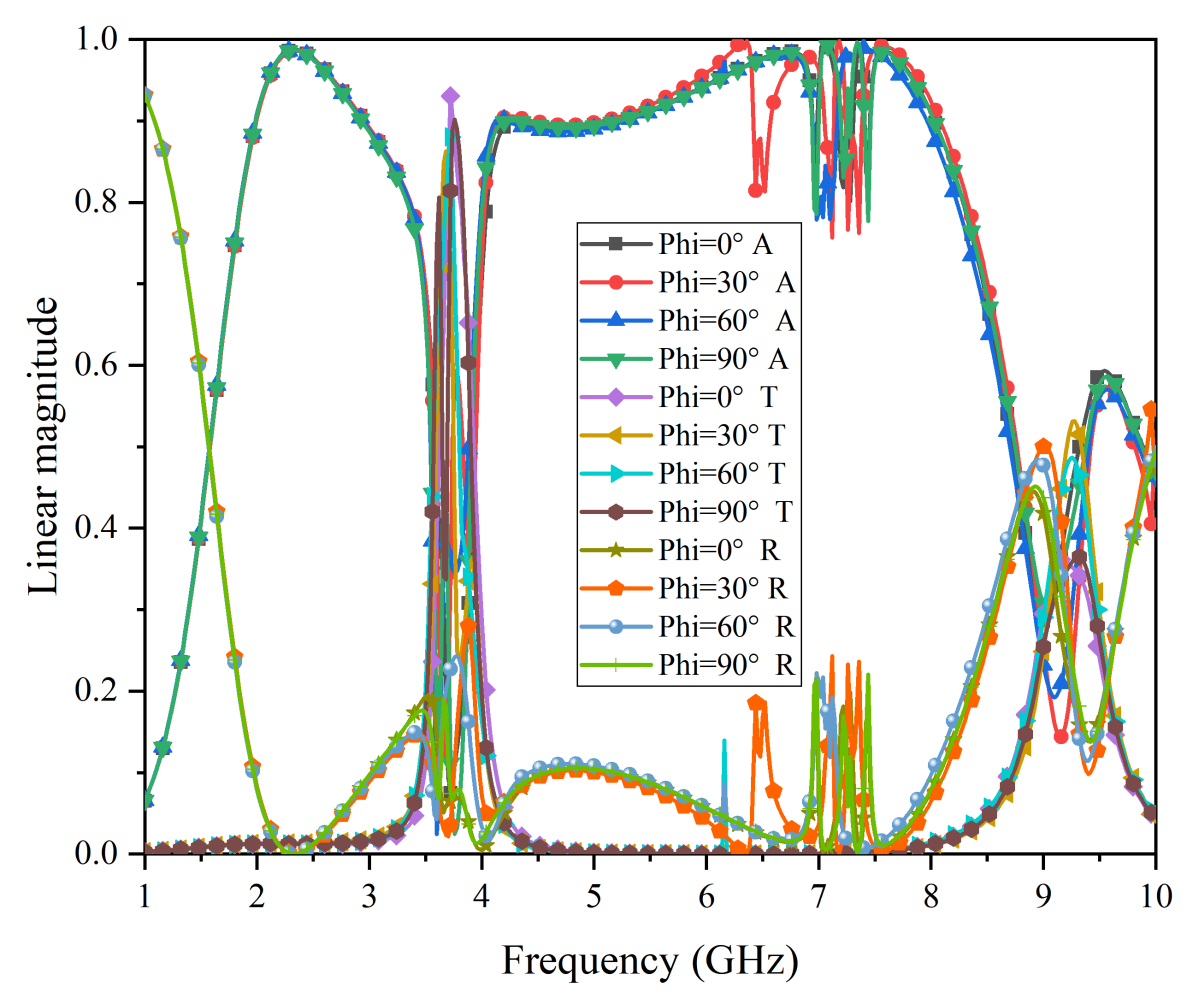
Simulation results for polarization stability for the proposed FSR.

Next, Figure 12 shows the simulation results in different polarizations under normal incidence for the proposed FSR. As can be seen from Figure 12, when the polarization angles are at 0°, 30°, 60°, and 90°, the structural performance remains basically consistent. Despite slight frequency deviations, both the transmission and absorption properties are still maintained well, indicating that this FSR structure possesses good polarization- insensitive characteristics.

**Figure 13:**
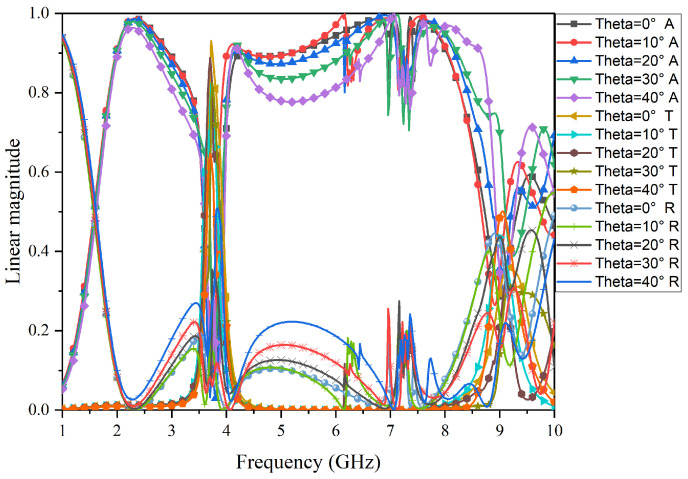
Simulation results for angular stability in TE-polarization for the proposed FSR.

**Figure 14:**
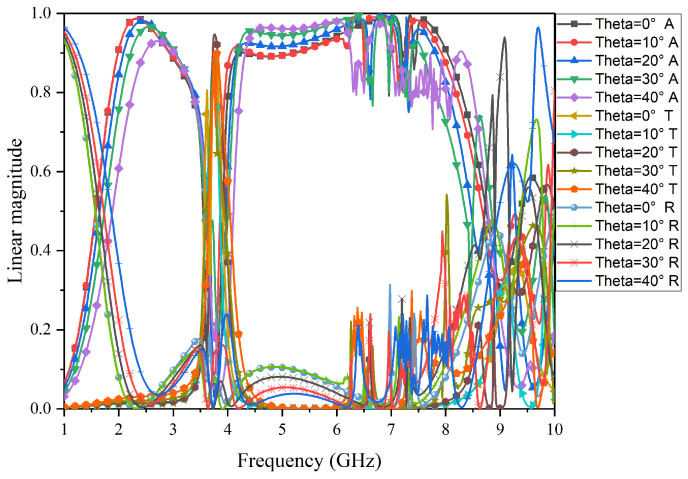
Simulation results for angular stability in TM-polarization for the proposed FSR.

The performance of FSR under an oblique incident was simulated and is shown in Figure 13 and Figure 14. Specifically, Figure 13 shows the results for TE-polarization. As the incidence angle increases to 40°, the transmission performance remains unchanged, and the bandwidth and absorption rate of the low-frequency absorption band and high-frequency band decrease gradually. Figure 14 shows the results for TM-polarization. As the incidence angle increases to 40°, the bandwidth of the transmission band gradually decreases, and the bandwidth and absorption rate of the low-frequency absorption band also gradually decrease, while the bandwidth of the high-frequency absorption band gradually decreases, but the absorption rate gradually increases. Although there are deviations, the overall filtering characteristics of the structure are not affected.

## 4. Experimental Characterization

**Figure 15:**
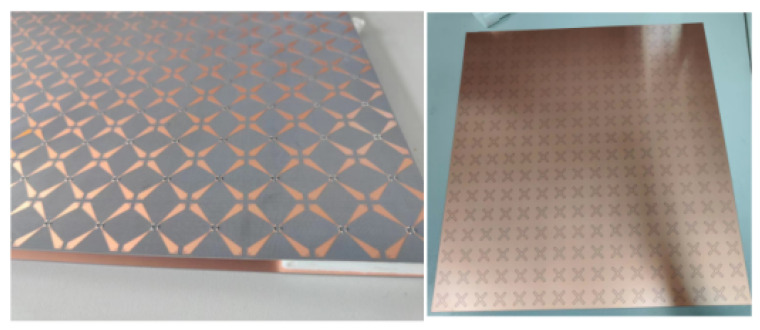
The prototype of the proposed FSR.

The prototype of the broadband rasorber designed in this study was processed to verify the performance of the rasorber. The finished product is shown in Figure 15. The overall size of the template is 400 mm × 400 mm × 13 mm, including 16 × 16 unit cells. The left side is the upper surface of the lossy layer, the right side is the cross-ring slot of the bandpass layer, and the middle is a dielectric substrate with a dielectric constant of 2.65 and an air layer supported by foam.

**Figure 16:**
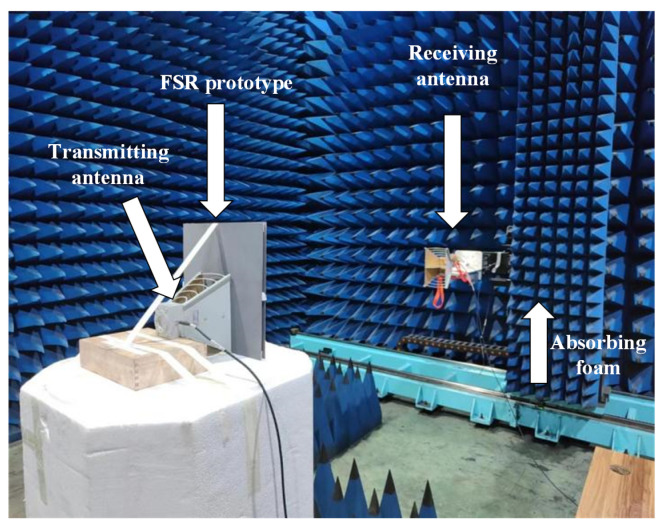
Test environment.

The electromagnetic characteristics of the prototype were measured in a microwave anechoic chamber. The test environment is shown in Figure 16. The prototype was fixed against the foam pier, the prototype was placed vertically, the transmitting antenna and the receiving antenna were on the same side, and the center of the transmitting antenna and the receiving horn antenna, as well as the geometric center of the prototype, were at the same level. The antenna was covered with wave-absorbing materials. To read the measured data, the receiving and transmitting antennas are connected through the RF connection line and the vector network analyzer, and then the measuring company integrates MATLAB R2023b into the measuring system for the final reading and data processing.

**Figure 17:**
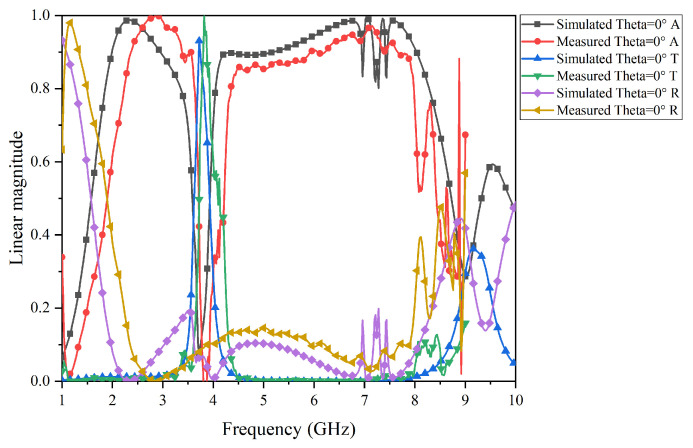
Comparison of simulation results and test results under TE-polarization in normal incidence.

**Figure 18:**
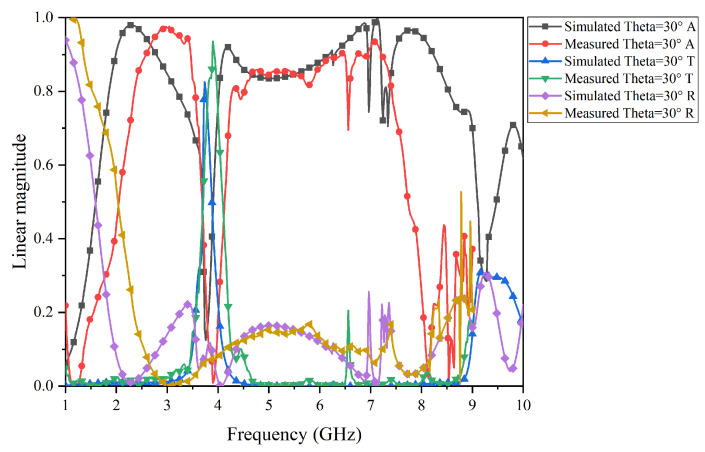
Comparison of simulation results and test results under TE-polarization in a 30° oblique incidence.

**Figure 19:**
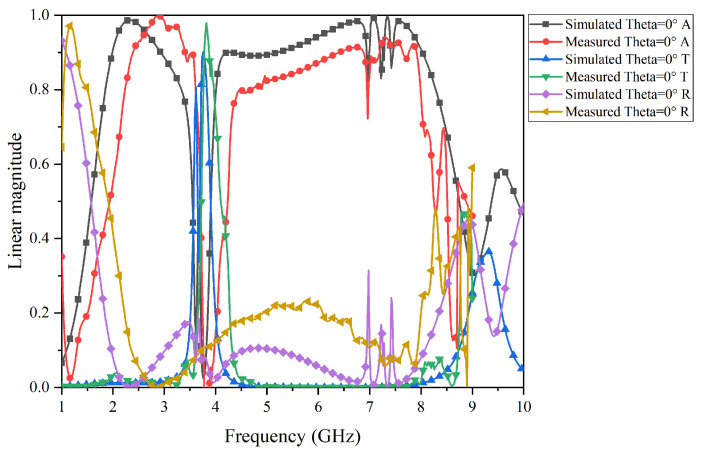
Comparison of the simulation results and test results under TM-polarization in normal incidence.

**Figure 20:**
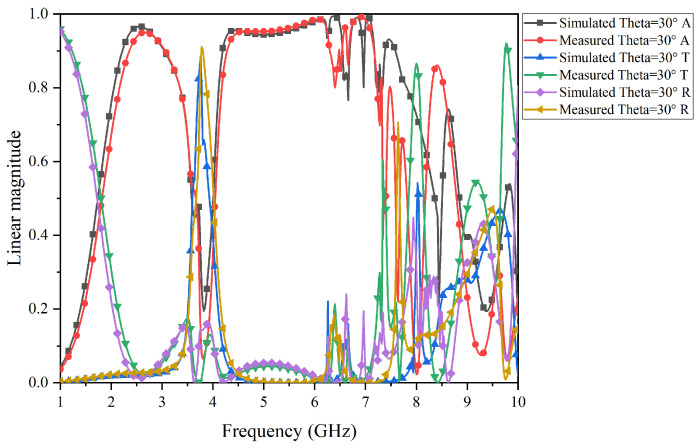
Comparison of simulation results and test results under TM-polarization in a 30° oblique incidence.

The test results of the prototype FSR are shown in Figure 17, Figure 18, Figure 19 and Figure 20. Specifically, Figure 17 and Figure 18 present the comparison curves between the test results and simulation results in normal incidence and 30° oblique incidence under TE-polarization. Figure 19 and Figure 20 present the comparison curves between the test results and simulation results in normal incidence and 30° oblique incidence under TM-polarization. From Figure 16 and Figure 17, it can be seen that the test results are highly consistent with the simulation results, further demonstrating that the FSR structure has excellent filtering performance and high-stability characteristics. The slight errors in the test process are mainly caused by environmental errors and instrument errors. Moreover, a comparison of this work with previously reported structures is given in Table 2.

## 5. Conclusions

In this article, a miniaturized and highly stable FSR incorporating an embedded transmission window is proposed. The resonance frequency and filtering performance of FSR remain highly consistent at different angles and under different polarizations. This FSR shows an embedded transmission window from 3.63 GHz to 3.80 GHz and has a transmission rate of 93% at 3.72 GHz. Moreover, both sides of the transmission band, namely, 1.86–3.35 GHz and 3.99–8.28 GHz, have an absorption rate of more than 80%, and the absorption bandwidth on both sides of the transmission band are all more than 50%. In addition, this structure exhibits excellent miniaturization performance such that the length and width of a unit are only $0.31\lambda_{T}\times 0.31\lambda_{T}$, where $\lambda_{T}$ represents the wavelength corresponding to the center frequency of the transmission band. The measured results are basically consistent with the simulation results. With its excellent filtering characteristics, this FSR can be applied in the field of stealth technology and electromagnetic compatibility.

## Figures and Tables

**Table 1:** The detailed structural parameters of the proposed FSR.

Parameter	Value (mm)	Parameter	Value (mm)	Parameter	Value (mm)
*L*	25.0	W4	0.2	L1	3.0
W1	3.0	W5	0.4	L2	15.3
W2	1.0	W6	2.0	L3	1.6
W3	0.1	W7	0.5		

**Table 2:** Performance comparison with existing designs.

Ref.	Absorption Bandwith	Transmission Rate	Unit Cell	Polarization	Angle
[19]	3.68–7.26 GHz (65.5%) 12.30–14.20 GHz (14.3%)	9.6 GHz (97%)	0.48$\lambda_{T}$	Dual	N.A.
[20]	1.92–3.73 GHz (64.1%) 7.41–9.34 GHz (23.0%)	5.74 GHz (91%)	1.15$\lambda_{T}$	Dual	35°
[21]	4.00–8.50 GHz (72.0%) 11.20–14.60 GHz (26.4%)	10.25 GHz (95%)	0.48$\lambda_{T}$	Dual	30°
[22]	6.26–9.58 GHz (41.9%) 11.46–14.06 GHz (20.4%)	10.4 GHz (88%)	0.62$\lambda_{T}$	Single	30°
[23]	1.83–4.06 GHz (75.7%) 4.56–6.44 GHz (34.1%)	4.3 GHz (97%)	0.23$\lambda_{T}$	Dual	40°
[24]	2.57–4.98 GHz (63.8%) 8.32–8.8 4GHz (6.0%)	6.6 GHz (90%)	0.44$\lambda_{T}$	Dual	45°
This paper	1.86–3.35 GHz (57.2%) 3.99–8.28 GHz (69.9%)	3.72 GHz (93%)	0.31$\lambda_{T}$	Dual	40°

## Data Availability

The data are contained within in the article.

## References

[1] Bai J., Yang Q., Liang Y., Gao X. (2022). Broadband Frequency Selective Rasorber Based on Spoof Surface Plasmon Polaritons. Micromachines.

[2] Fan C., Duan K., Chen K., Jiang T., Zhao J., Feng Y. (2023). Actively tunable rasorber with broadband RCS reduction and low infrared emissivity. Opt. Express.

[3] Priyanka S., Mohanty S.S., Alegaonkar P.B., Baskey H. (2023). Design and Manufacturing of a Hexapattern Frequency Selective Surface Absorber for Aerospace Stealth Application. ACS Appl. Mater. Interfaces.

[4] Qin T., Huang C., Cai Y., Lin X. (2023). Dual-Band Frequency Selective Surface with Different Polarization Selectivity for Wireless Communication Application. Sensors.

[5] Ruan J., Meng Z., Zou R., Cai F., Pan S. (2022). Miniaturized Frequency Selective Surface for 6G Communication. Micromachines.

[6] Niaz M.W., Yin Y., Zheng S., Zhao L., Chen J. (2020). Design and Analysis of an Ultraminiaturized FSS Using 2.5-D Convoluted Square Spirals. IEEE Trans. Antennas Propag..

[7] Wei P.-S., Chiu C.-N., Chou C.-C., Wu T.-L. (2020). Miniaturized Dual-Band FSS Suitable for Curved Surface Application. IEEE Antennas Wirel. Propag. Lett..

[8] Mahmoodi M., VanZant L., Donnell K.M. (2020). An Aperture Efficiency Approach for Optimization of FSS-Based Sensor Resolution. IEEE Trans. Instrum. Meas..

[9] Zhang Y., Li B., Zhu L., Tang Y., Chang Y., Bo Y. (2018). Frequency Selective Rasorber with Low Insertion Loss and Dual-Band Absorptions Using Planar Slotline Structures. IEEE Antennas Wirel. Propag. Lett..

[10] Kong X., Kong L., Jiang S., Wang X., Zou Y., Xing L. (2021). Low-Profile and Dual-Polarization Water-Based Frequency Selective Rasorber with Ultrawideband Absorption. IEEE Antennas Wirel. Propag. Lett..

[11] Qian G., Zhao J., Ren X., Chen K., Jiang T., Feng Y., Liu Y. (2019). Switchable Broadband Dual-Polarized Frequency-Selective RasorberAbsorber. IEEE Antennas Wirel. Propag. Lett..

[12] Liu N., Sheng X., Zhao X. (2020). Design of Dual-Polarized Frequency Selective Rasorber With Two Independent Transmission Windows Using Multi-Resonators. IEEE Access.

[13] Zhang X., Wu W., Huang L., Ma Y., Yuan N. (2019). Design of Dual-Absorptive-Bands Frequency Selective Rasorber with Minkowski Loop Arrays. IEEE Antennas Wirel. Propag. Lett..

[14] Kwon D.H. Lossless Tensor Surface Invisibility Cloaks Utilizing Surface Waves. Proceedings of the 2018 12th International Congress on Artificial Materials for Novel Wave Phenomena (Metamaterials).

[15] Idris F.M., Hashim M., Ismayadi I., Idza I.R., Manap M., Shafie M.S.E. (2013). Broadening of EM Energy-Absorption Frequency Band by Micrometer-to-Nanometer Grain Size Reduction in NiZn Ferrite. IEEE Trans. Magn..

[16] Costa F., Monorchio A. (2012). A Frequency Selective Radome with Wideband Absorbing Properties. IEEE Antennas Wirel. Propag. Lett..

[17] Hu Y., Liu S., Kong X., Mao C. (2017). Low windage resistance frequency-selective rasorber based on windmill-shape coupling line arrays. Electron. Lett..

[18] Omar A.A., Shen Z., Huang H. (2017). Absorptive Frequency-Selective Reflection and Transmission Structures. IEEE Trans. Antennas Propag..

[19] Wang L., Liu S., Kong X., Zhang H., Yu Q., Wen Y. (2020). Frequency-Selective Rasorber with a Wide High-Transmission Passband Based on Multiple Coplanar Parallel Resonances. Micromachines.

[20] Zhang X., Wu W., Ma Y., Wang C., Li C., Yuan N. (2019). Design Dual-Polarization Frequency Selective Rasorber Using Split Ring Resonators. IEEE Access.

[21] Yu Q., Liu S., Monorchio A., Kong X., Wen Y., Huang Z. (2019). A Miniaturized High-Selectivity Frequency Selective Rasorber Based on Subwavelength Resonance and Interdigital Resonator. IEEE Antennas Wirel. Propag. Lett..

[22] Guo M., Guo T., Cheng Q., Zheng Y., Fu Y. (2021). Frequency Selective Rasorber with Anisotropic Transmission Band. IEEE Antennas Wirel. Propag. Lett..

[23] Xiu X., Che W., Yang W., Han Y., Xue Q. (2020). A Highly Selective Rasorber Based on Second-Order Resonance. IEEE Antennas Wirel. Propag. Lett..

[24] Parameswaran A., Kundu D., Sonalikar H.S. (2021). A Dual-Polarized Wideband Frequency-Selective Rasorber with Low in-Band Insertion Loss and High Oblique Incidence Stability. IEEE Trans. Electromagn. Compat..

